# An AS-OCT image dataset for deep learning-enabled segmentation and 3D reconstruction for keratitis

**DOI:** 10.1038/s41597-024-03464-0

**Published:** 2024-06-13

**Authors:** Yiming Sun, Nuliqiman Maimaiti, Peifang Xu, Peng Jin, Jingxuan Cai, Guiping Qian, Pengjie Chen, Mingyu Xu, Gangyong Jia, Qing Wu, Juan Ye

**Affiliations:** 1 Eye Center, The Second Affiliated Hospital, School of Medicine, Zhejiang University, Zhejiang Provincial Key Laboratory of Ophthalmology, Zhejiang Provincial Clinical Research Center for Eye Diseases, Zhejiang Provincial Engineering Institute on Eye Diseases, Hangzhou, Zhejiang China; 2 College of Computer Science, Hangzhou Dianzi University, Hangzhou, China; 3 School of Mathematical Sciences, Zhejiang University, Hangzhou, Zhejiang China; 4 College of Media Engineering, Communication University of Zhejiang, Hangzhou, China

**Keywords:** Corneal diseases, Infectious diseases

## Abstract

Infectious keratitis is among the major causes of global blindness. Anterior segment optical coherence tomography (AS-OCT) images allow the characterizing of cross-sectional structures in the cornea with keratitis thus revealing the severity of inflammation, and can also provide 360-degree information on anterior chambers. The development of image analysis methods for such cases, particularly deep learning methods, requires a large number of annotated images, but to date, there is no such open-access AS-OCT image repository. For this reason, this work provides a dataset containing a total of 1168 AS-OCT images of patients with keratitis, including 768 full-frame images (6 patients). Each image has associated segmentation labels for lesions and cornea, and also labels of iris for full-frame images. This study provides a great opportunity to advance the field of image analysis on AS-OCT images in both two-dimensional (2D) and three-dimensional (3D) and would aid in the development of artificial intelligence-based keratitis management.

## Background & Summary

Infectious keratitis is among the major causes of corneal opacity, which is considered one of the top 5 causes of global blindness and moderate to severe vision impairment^1,2^. It is a great burden to the socioeconomics since an estimated 5.5 million people are bilaterally blind and 6.2 million people are unilaterally blind resulting from corneal opacity^3^. To avoid severe complications resulting in vision impairment or even blindness, infectious keratitis requires prompt attention as it can progress rapidly. The disease is diagnosed and managed typically based on slit-lamp observation, nevertheless, it cannot characterize the severity of stromal inflammation and other changes of internal structures in the cornea during monitoring the progress of corneal pathologies, thus may lead to unreversible outcomes^4^.

The emergence of AS-OCT enables the accurate analysis and monitoring of the progress of corneal pathologies in patients with keratitis by providing cross-sectional images^5–7^. This optical device is non-invasive and its multiple 360-degree 2D frames also enable a 3D view of the anterior chamber in high resolution. However, clinical evaluation typically selects one frame from the whole view, due to its time-consuming manual analysis process, which may result in the missing and waste of information. With the development of artificial intelligence in ophthalmology, automatic analysis techniques based on deep learning have been applied to several modalities of ophthalmic images^8,9^. The rising applications of AS-OCT image analysis algorithms mainly focused on corneal segmentation, anterior segment biometry, and angle closure glaucoma, with barely any studies on keratitis. Also, the majority of these studies based on AS-OCT images utilized relatively limited datasets, and most of these datasets are not open access, not to mention annotated images which are impractical and time-costly in settings such as busy clinics^10^.

**Fig. 1:**
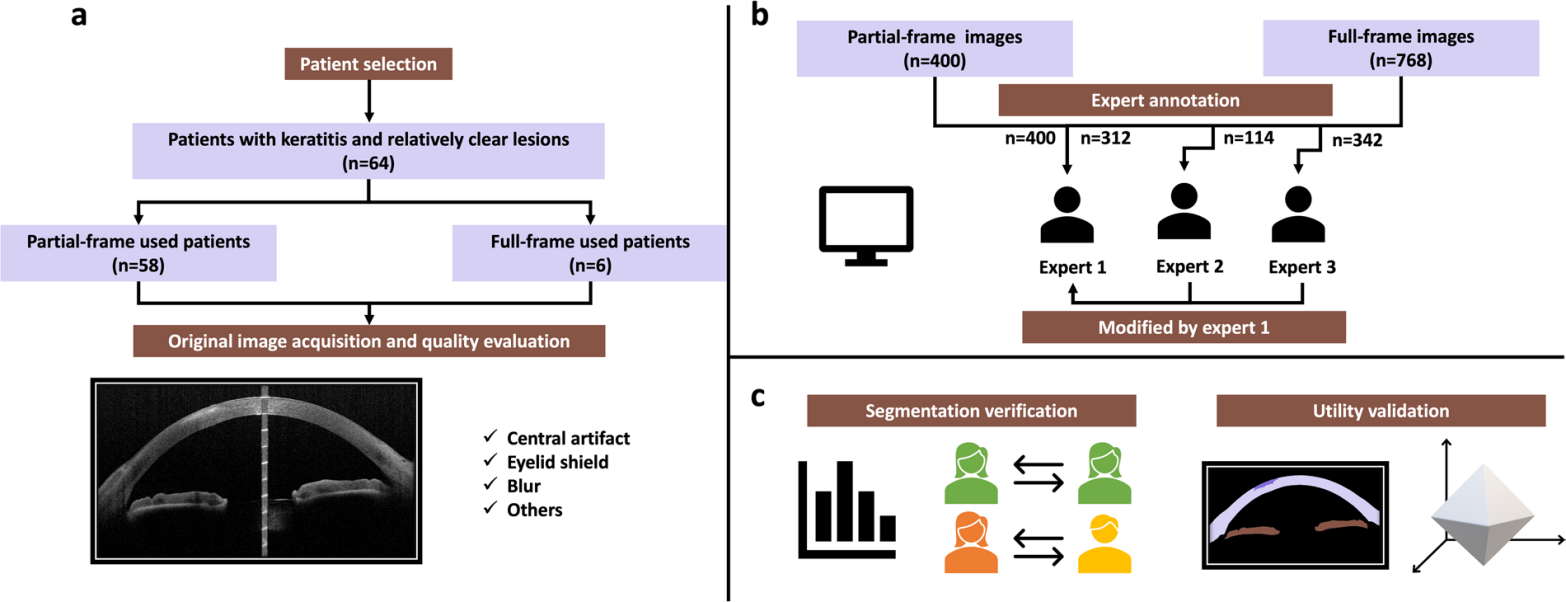
The pipeline overview of the work. (**a**) Dataset preparation. (**b**) Experts’ annotation and modification. (**c**) Dataset validation.

For this reason, the proposed AIDK dataset, including a large number of images with expert annotation, intends to advance the field of AS-OCT image analysis methods for patients with keratitis, both in 2D segmentation and 3D analysis. Figure 1 illustrates the major workflow of the AIDK data set, including dataset preparation, experts’ annotation and modification, and dataset validation.

## Methods

### Ethics declaration

The Ethics Committee of the Second Affiliated Hospital of Zhejiang University, College of Medicine, approved this study (IR2021001176). All methods adhered to the tenets of the Declaration of Helsinki. The data was collected from patients via routine medical treatment and then obtained from medical facilities retrospectively. Also, the data doesn’t contain direct identifiers. All participants’ names have been removed and their IDs have been rearranged to anonymize identifiable information. Therefore, the Ethics Committee of the Second Affiliated Hospital of Zhejiang University, College of Medicine has issued a waiver of consent.

### Patient selection and image acquisition

This study retrospectively included 64 patients diagnosed with keratitis, between September 2018 and March 2023 at the Eye Center, the Second Affiliated Hospital of Zhejiang University, College of Medicine, China. Patients with unclear corneal lesions or poor image quality that could affect the visibility of anterior segment characteristics in AS-OCT were excluded. There were no selection criteria on keratitis cause. The population profiles are summarized in Table 1. The mean and standard deviation of participant age was 54.6 ± 19.3 years (ranging from 4 to 92 years).

**Table 1:** Population profiles of the AIDK dataset. FFD: Full-frame dataset, PFD: Partial-frame dataset.

	Total	FFD	PFD
**Number of patients**	64	6	58
**Age in years**	54.6 ± 19.3 [92, 4]	53.0 ± 19.2 [86,34]	54.8 ± 19.8 [94,2]
**Gender**			
Male	44 (68.7%)	3 (50.0%)	41 (70.7%)
Female	20 (31.3%)	3 (50.0%)	17 (29.3%)

Anterior segment imaging was carried out by swept-source Casia SS-1000 AS-OCT (Tomey Corporation, Nagoya, Japan), with the scanning beam running through the center of the cornea. A total of 1168 original AS-OCT images in BMP format were obtained directly from the software of the machine without any image preprocessing. The NO.1–5, 15–70 images were collected from the first machine (the same type as the second machine), with a resolution of 1657 × 1000 pixels. Other images were collected from the second machine, with a resolution of 1723 × 1000 pixels. The ratio of length per pixel of the images collected from the first and second machines is 49:47. Then the images were split into 2 categories: partial-frame dataset (n = 400, 58 patients) and full-frame dataset (n = 768, 6 patients). The partial-frame dataset contains each patient’s partial frames (selected from 128 frames per person), and the full-frame dataset refers to the total 128 frames of each patient were included. The partial frames were selected based on the following criteria: (1) Without massive artifacts affecting the critical information. (2) Exhibit relatively clear lesion areas the corneas.

### Annotations in 2D and 3D

The lesion, cornea, and iris of all slices from the full-frame dataset were manually contoured by experts, enabling reconstruction in 3D. Also, the lesion and cornea of 2D slices from the partial-frame dataset were annotated. The diagnosis and treatment for keratitis mainly focus on the cornea and lesion area, therefore the “iris” label is not available for 2D slices. Nevertheless, to enable the reconstruction of a relatively intact anterior chamber, the study provided the “iris” label for the full-frame dataset.

In the initial annotation step, the contour of the lesion, cornea in all images, and iris in full-frame images were annotated by 3 experts using Labelme^11^ as follows: (1) Expert 1 (E1) ophthalmologist with 5 years of experience: Partial-frame dataset (n = 400) and 312 images from the full-frame dataset. (2) Expert 2 (E2) ophthalmologist with 3 years of experience: 114 images from the full-frame dataset. (3) Expert 3 (E3) ophthalmologist with 1 year of experience: 342 images from the full-frame dataset. Then, all annotations were checked and modified by expert 1. Additionally, the partial-frame images were further checked and modified by an expert specialized in ocular surface with 10 years of practice experience. The consolication process aims to eliminate mislabeling and to guarantee agreement between different experts. Therefore, the consolication process includes the following steps: (1) Checking if the annotated polygons are correctly matched with each label. If the polygons are not correctly matched with each label, then correction would be made directly. (2) Checking whether the lesion exists on each image. (3) Ensuring the boundaries of each polygon are properly annotated. If there is a disagreement on whether the lesion exists or on the boundaries of polygons, a more experienced ophthalmologist would be consulted to make the final decision together. Then the annotations and labels would be removed and reannotated if needed. Normally, due to the relatively high image contrast, the boundaries are relatively clear with negligible difference and no need to modify, only a few lesions are controversial.

**Fig. 2:**
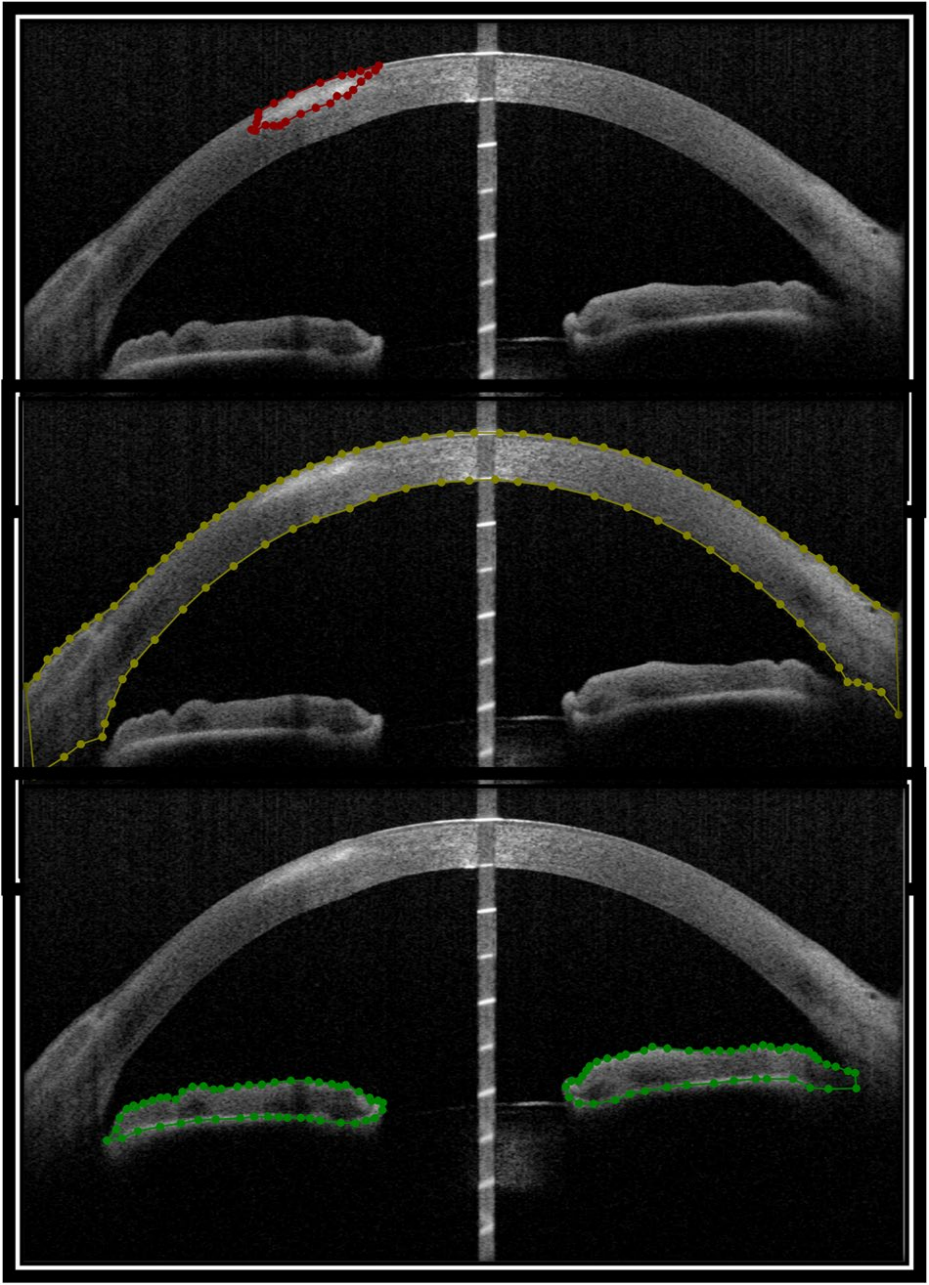
Examples of the lesion, cornea, and iris annotations provided by an expert.

It is noteworthy that before annotations, all experts agreed on the annotation criteria as below (Fig. 2), and were trained and tested on certain example images: (1) Lesion: The hyper-reflective area that corresponded to the clinical infiltration. (2) Cornea: Cornea and anterior sclera which are visible in AS-OCT images with no deformation caused by imaging artifact. (3) Iris: The part of the iris with clear boundaries in AS-OCT images.

## Data Records

The AIDK dataset is available at the public figshare repository^12^. Features of the dataset were summarized in Table 2 and the file “Demographics of participants.xlsx”. The 2 categories of images are separated into 2 folders, named “Partial-frame_Dataset” and “Full-frame_Dataset” respectively. In each folder, original AS-OCT images in the BMP format are provided in the “Original_AS-OCT_Images” folder named as “n.bmp”, and corresponding annotated files in the JSON format are provided in the “Experts_Annotations” folder named as “n.json”.

**Table 2:** Features of the AIDK dataset. FFD: Full-frame dataset, PFD: Partial-frame dataset.

Particulars	AIDK data set		
	Total	FFD	PFD
Total number of AS-OCT images	1168	768	400
Imaging devices used	Tomey (Casia SS-1000; image resolution: 1657 × 1000 pixels & 1723 × 1000 pixels)		
Exclusion of poor-quality images	✓	✓	✓
Number of experts participated in annotations	3	3	2
Lesion outlining provided by experts	✓	✓	✓
Cornea outlining provided by experts	✓	✓	✓
Iris outlining provided by experts		✓	
The annotations verified by experts	✓	✓	✓
Demographics of participants	✓	✓	✓

The folder “Original_AS-OCT_Images” in the “Partial-frame_Dataset” contains 400 partial-frame AS-OCT images, with the “n” ranging from 1 to 400 denoting the nth sample. The NO.1–5, 15–70 images have a resolution of 1657 × 1000 pixels, and other images have a resolution of 1723 × 1000 pixels. The ratio of length per pixel of the two size images is 49:47. The corresponding folder “Experts_Annotations” contains the same size of files, and the annotations include the lesion labeled as “Lesion” and the cornea labeled as “Cornea”.

The folder “Original_AS-OCT_Images” in the “Full-frame_Dataset” contains 768 full-frame AS-OCT images, with the “n” ranging from 401 to 1168 denoting the nth sample. The files named “401” to “528”, “529” to “656”, “657” to “784”, “785” to “912”, “913” to “1040”, and “1041” to “1168” respectively represent the full-frame scan (128 frames/person) of one of the 6 patients. The images have a resolution of 1723 × 1000 pixels. The corresponding folder “Experts_Annotations” contains the same size files, and the annotations include the lesion labeled as “Lesion”, the cornea labeled as “Cornea” and the iris labeled as “Iris”.

The file “Demographics of participants.xlsx” consists of a 6-columns sheet. From left to right, the 6 columns represent “Patient ID”, “Image ID”, “Age at acquisition”, “Acquired eye”, “Biological sex”, and “Mostly Labeled by Expert1/2/3” respectively.

## Technical Validation

### Image quality verification

**Fig. 3:**
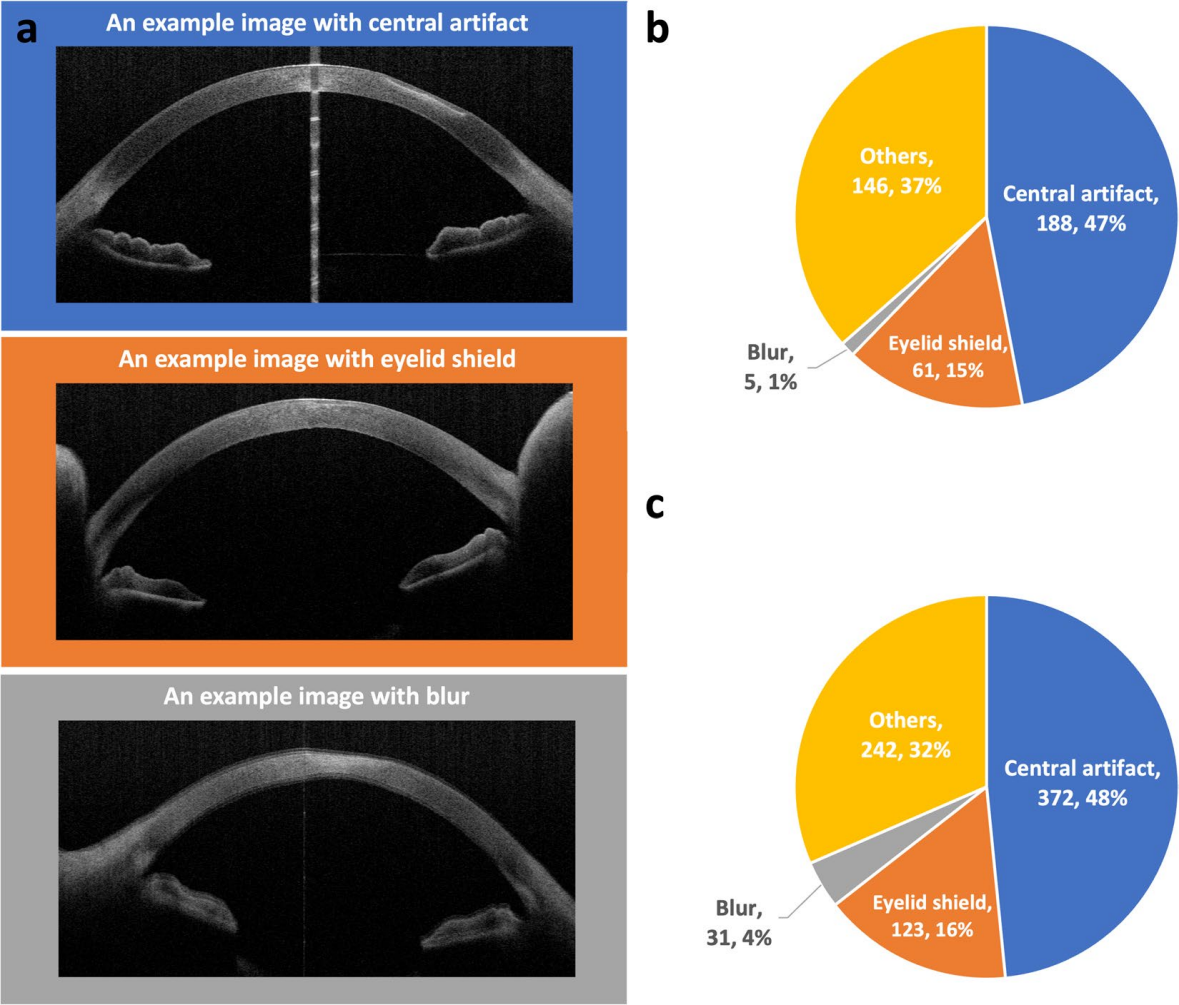
Quality evaluation of the AIDK dataset. (**a**) Examples with certain defects. (**b**) The quality evaluation of partial-frame images (n = 400). (**c**) The quality evaluation of full-frame images (n = 768).

Some certain artifacts can occur in AS-OCT images, which might affect the training process of deep learning methods. There are several studies for posterior segment OCT image quality assessments^13–16^. However, the criteria to assess the quality of AS-OCT images are different. In recent years, methods to assess AS-OCT image quality have been developed^17,18^, nevertheless, they are not specific to cornea diseases, and the approach still needs validation. Here, three main artifacts possibility relevant were evaluated in this study (Fig. 3a), defined as (1) Central artifact: The central hyper-reflective or hypo-reflective area caused by the central beam during imaging, (2) Eyelid shield: The occlusion of the eyelid resulting in the missing or deformation of the anterior chamber angle area, (3) Blur: Artifacts resulting from movement of the eye.

All images were evaluated, and Fig. 3 shows the distribution of image quality in both the partial-frame dataset and the full-frame dataset. Central artifact occurs in 47% of the partial-frame dataset and 48% of the full-frame dataset. This can be addressed via approaches to detect and remove the central artifact proposed by previous studies^19^. 15% of images in the partial-frame dataset and 16% of images in the full-frame dataset display eyelid shields but without occlusion of the cornea. This suggests that most of the images can also be used for anterior chamber angle area analysis. Less than 5% of images in the partial-frame dataset (1%) and the full-frame dataset (4%) show the artifact of blur.

### Segmentation evaluation

The annotations were verified by evaluating the consistency of annotations between the same and different annotators (E1 vs. E1, E1 vs. E2, E1 vs. E3, E2 vs. E3). To make comparisons, the same 20 images randomly selected from the AIDK dataset were manually segmented by the three experts. Then, the mean intersection over union (IoU) and Dice coefficient were calculated between different annotators. Similarly, the same 20 images were segmented once more by Expert 1, to verify the consistency of the same annotator. Also, the intraclass correlation coefficient (ICC) was used to indicate the degree of agreement between the segmentations of the 3 experts.

**Fig. 4:**
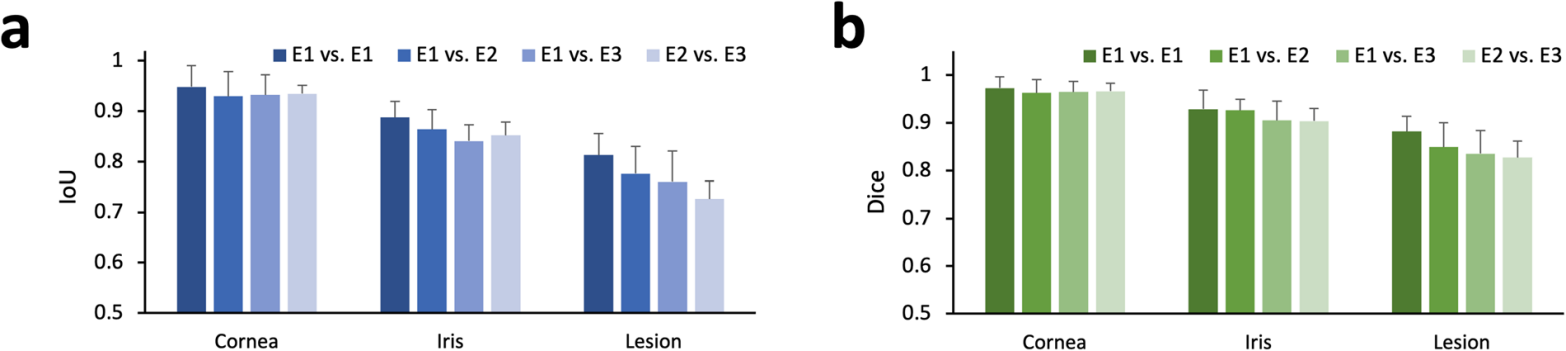
Segmentation verification of the AIDK dataset. (**a**) The IoU of the manually segmented cornea, iris, and lesion between different annotators. (**b**) The Dice score of the manually segmented cornea, iris, and lesion between different annotators.

Figure 4 shows the IoU and Dice coefficient between segmentations. The annotation of the cornea shows high consistency (all mean IoU > 0.93 and all mean Dice scores > 0.96). Even though AS-OCT has limitations in imaging iris, the annotation of the iris still shows relatively good consistency (all mean IoU > 0.84 and all mean Dice scores > 0.90). The annotations of lesion show acceptable consistency (all mean IoU > 0.73 and all mean Dice scores > 0.83). The ICCs between different areas labeled by the 3 experts were 0.897 for the cornea, 0.900 for the iris, and 0.708 for the lesion. This indicated that there is excellent agreement between different annotations of the cornea and the iris, as well as substantial agreement for annotations of the lesion^20^. In some cases, the infiltration of corneal lesions can sometimes make the lesion boundaries less clear, which is relatively common in clinical settings. This may result in lower IoU, Dice scores, and ICCs in corneal lesions, in comparison to that of cornea and iris.

### Dataset utility

The 2 categories of datasets enable utility in both 2D analysis and 3D reconstruction of keratitis lesions and corresponding anterior chambers. Currently, we are working on a 2D analysis deep-learning-based method of AS-OCT images with keratitis lesions. We trained and tested the segmentation module based on the AIDK dataset and along with another supplement dataset. The module was developed on an improved U-Net architecture^21–26^ and achieved a Dice score of 0.79 for initial segmentation on the keratitis lesion. This would be specifically demonstrated in our future work. Also, the full-frame dataset provides manual contouring of all slices in full 3D regions of interest. This can increase the accuracy of the segmentation in the third dimension since 3D data are generally not annotated in full 3D due to the extremely high contouring workload^27,28^. We are also working on the 3D reconstruction of the keratitis-specific cornea and anterior chamber based on AS-OCT images, and have acquired initial results, which will also be demonstrated in our future work.

## Usage Notes

The AIDK dataset presented in this paper can be downloaded through the link mentioned above. Users should properly acknowledge the contributions and cite this article.

## Data Availability

No custom code was used.
